# Dysfunctional Electron Transport Chain Assembly in COXPD8

**DOI:** 10.3390/jcdd12080318

**Published:** 2025-08-20

**Authors:** Gisela Beutner, Heidie L. Huyck, Gail Deutsch, Gloria S. Pryhuber, George A. Porter Jr.

**Affiliations:** 1 Department of Pediatrics-Division Cardiology, University of Rochester Medical Center, Rochester, NY 14642, USA; gisela_beutner@urmc.rochester.edu; 2 Department of Pediatrics-Division of Neonatology, University of Rochester Medical Center, Rochester, NY 14642, USA; heidie_huyck@urmc.rochester.edu (H.L.H.); gloria_pryhuber@urmc.rochester.edu (G.S.P.); 3 Department of Pathology, Seattle Children’s Hospital and University of Washington Medical Center, Seattle, WA 98101, USA; deutsg@uw.edu

**Keywords:** mitochondrial disease, COXPD8, electron transport chain, mitochondrial supercomplexes, hypertrophic cardiomyopathy

## Abstract

Combined oxidative phosphorylation deficiency type 8 (COXPD8) is an autosomal recessive mitochondrial disorder caused by a mutation of the nuclear encoded mitochondrial alanyl-tRNA synthetase gene (AARS2). Clinical manifestations of COXPD8 include lethal infantile hypertrophic cardiomyopathy, pulmonary hypoplasia, generalized muscle weakness, and neurological involvement. We report a patient with COXPD8 caused by two mutations in the AARS2 gene. The c.1738 C>G mutation has not been previously reported, while the c.2872 C>T mutation has been associated with pulmonary hypoplasia and hypertrophic cardiomyopathy. Cardiac tissue, obtained through the LungMAP program, showed that, compared to other patients of similar ages, these two mutations affect not only the assembly of functional monomeric complexes (Cx) I and IV of the electron transport chain (ETC) but also limit the formation of respiratory supercomplexes. This patient had altered expression of some ETC proteins but normal expression of several enzymes of the tricarboxylic acid cycle. We also show that one of the control/comparison patients had an undiagnosed ETC Cx IV deficiency. In conclusion, our data demonstrate that the two mutations of the AARS2 gene are associated with failed assembly of Cx I and Cx IV and reduced formation of respiratory supercomplexes of the ETC, likely leading to acute bioenergetic stress.

## 1. Introduction

Combined oxidative phosphorylation deficiency type 8 (COXPD8) is an autosomal recessive disorder caused by a mutation of a mitochondrial alanyl-tRNA synthetase gene (AARS2) encoded on chromosome 6 [1]. Generally, the function of the mitochondrial aminoacyl-tRNA synthetase is to attach a specific amino acid to one of its cognate tRNAs to form an aminoacyl-tRNA [2]. Human mitochondrial aminoacyl-tRNA synthetases are encoded in the nucleus, synthesized in the cytosol, then imported into mitochondria via a mitochondrial targeting sequence, which is cleaved upon entry into mitochondria [2]. The mammalian mitochondrial genome, transmitted exclusively through the female germ line, contains 37 genes coding for two rRNAs, 22 tRNAs and 13 proteins [3]. Together with 84 additional nucleus-encoded proteins, these 13 mitochondrial-encoded proteins form the complexes of the electron transport chain (ETC) [3]. Thus, mitochondrial aminoacyl-tRNA synthetases are central to cellular energy production.

Despite ubiquitous expression, autosomal recessive mitochondrial aminoacyl-tRNA synthetase disorders are associated with highly tissue- and cell-specific phenotypes that typically involve the central nervous system [2,4]. However, a recessively inherited variant of AARS2 was described in patients presenting with fatal infantile cardiomyopathy and multiple oxidative phosphorylation (OXPHOS) defects [1]. Clinical manifestations of COXPD8 include a lethal infantile hypertrophic cardiomyopathy, while skeletal muscle and the brain, but not the liver, may also be affected [1]. Pulmonary hypoplasia, generalized muscle weakness, and neurological involvement are additional characteristic features of COXPD8.

COXPD8 patients have deficiencies in complexes (Cx) I, III, and IV of the mitochondrial ETC in skeletal muscle [4] and in Cx I and IV in cardiac muscle and brain [1] by various methods. The monomers of Cx I, III and IV are known to assemble into supercomplexes for optimized functionality [5]. Here we report a patient with COXPD8 caused by two mutations in the AARS2 nuclear gene. The c.2872 C>T mutation was previously associated with pulmonary hypoplasia and hypertrophic cardiomyopathy and is considered likely pathogenic [6], while the c.1738 C>G mutation has not been reported and is considered a variant of unknown significance. We show that these mutations affect the expression of ETC complex proteins and assembly of functional monomeric complexes I and IV of the ETC, limit the formation of respiratory supercomplexes but have no apparent impact on the expression of several enzymes of the tricarboxylic (TCA) cycle. We compared the ETC complex proteins of the AARS2 affected infant with those of other “control/comparison” infants expected to have normal mitochondrial metabolism. Unexpectedly, we found that one of the control patients had an undiagnosed mitochondrial abnormality of Cx IV of the ETC.

## 2. Materials and Methods

### 2.1. Study Population

Heart tissues from one COXPD8 and five neonatal patients, who died from various causes between 1 day and 5 months of life (Table 1), were authorized for research and provided, with de-identified clinical data, through the Developmental Lung Molecular Atlas Program (LungMAP) Human Tissue Core (HTC) and BioRepository for Investigation of Neonatal Diseases of the Lung (BRINDL).

### 2.2. Case Presentation

The LungMAP HTC received the lungs and heart of a male patient who passed away on day of life (DOL) 51 with a genetic diagnosis of COXPD8. The infant was born at 38 5/7 weeks gestation by C-section. His birth weight was 3.6 kg, and he was 52 cm long and had a head circumference of 37 cm. His APGAR scores were 1/1/5/5 due to no respiratory effort, bradycardia, and flaccid hypotonia. Therapeutic whole-body cooling for hypoxic–ischemic encephalopathy (HIE) was instituted shortly after birth and continued for 72 h per protocol.

An echocardiogram on DOL 1 showed severe pulmonary hypertension with significant biventricular cardiac dysfunction and hypertrophy. A chest X-ray showed minimal expansion despite peak inspiratory and end-expiratory pressures of 27/17 mm Hg, raising concern for pulmonary hypoplasia. The infant was transitioned to high-frequency jet ventilation with better expansion, oxygenation and ventilation but severe mixed acidosis continued. By 8 h of life, veno-arterial extracorporeal membrane oxygenation (ECMO) was started, but poor oxygenation (requiring 100% O_2_ on the ECMO circuit) and lactic acidosis continued. The first and subsequent EEGs were consistent with moderate-to-severe hypoxic–ischemic encephalopathy.

On DOL 1, acidosis continued with severely elevated lactate and ammonia (244 micromol/L). Hepatic and renal function tests were otherwise normal. The patient received continuous renal replacement therapy for lactic acidosis beginning on DOL 2. Profound cardiac failure with cardiac hypertrophy was diagnosed on DOL 2, but by DOL 3, cardiac function was near normal and initial dysfunction was thought to be “cardiac stunning” on ECMO [7]. Elevated right ventricular pressure persisted with mild right ventricular hypertrophy. Seizures occurred but were thought to occur earlier in the course than typical after HIE. Head ultrasounds were normal.

The infant was treated with stress dose hydrocortisone until DOL 6, dobutamine and bicarbonate drips, and diuresis. An echocardiogram on DOL 12 showed two muscular ventricular septal defects and a patent foramen oval with left to right shunting and modest to no cardiomyopathy.

From DOL 2–6 lung expansion improved but remained abnormal. On DOL 9, removal of a mucus plug resulted in improved expansion, and ventilatory settings were able to be weaned so that stable permissive hypercapnia was achieved. Decannulation from ECMO was successful on DOL 12, and pressors were required to DOL 14. On DOL 15 a chest CT showed grossly normal cardiothymic size, grossly normal lung expansion, but extensive bilateral airspace disease with infiltrates and possible pneumonia; the patient continued to receive antibiotics and high frequency jet ventilation.

The infant had initial hyperglycemia followed by intermittent hypoglycemia requiring increased dextrose infusion. He was maintained on standard total parenteral nutrition and tolerated advancing feeds. The overall respiratory status improved and he was weaned to conventional ventilation with a nadir of 0.25 FiO_2_. However, successive acute episodes of respiratory decompensation, mixed and lactic acidosis, and worsening left ventricular hypertrophy and left ventricular outflow tract obstruction, led to a transition to comfort care on DOL 46.

The patient has a normal karyotype (46XY). A single nucleotide polymorphism microarray did not identify any DNA deletions or duplications. Whole exome sequencing revealed two mutations in the AARS2 nuclear gene:

1. Exon/Intron 22 c.2872c>T; p.Arg 958Ter, a heterozygous maternal, autosomal recessive mutation, consistent with that previously reported in a family of three siblings with hypertrophic cardiomyopathy [6]). The mutation was classified as likely pathogenic.
2. Exon/Intron 12 c.1738C>G; p.Arg580Gly, heterozygous paternal, autosomal recessive, with the same expected phenotype though not previously reported. Different missense mutations at this amino acid have been associated with fatal infantile cardiomyopathy, although the mutation was classified as of uncertain significance.

Based on the presence of these mutations, the patient was diagnosed with COXPD8 (OMIM:612035) and, beginning on DOL24 due to the genetic testing results, was treated with medium chain triglycerides, carnitine, and coenzyme Q10 as standard therapy in patients with presumed or apparent mitochondrial genetic defects

### 2.3. Comparison Infant Patients

The results of the biochemical experiments of the COXPD8 patient were compared to those from 5 patients (Table 1) that died within the first few months of life.

### 2.4. Tissue Biobank

Heart and lung organs were screened, accepted and accessioned into BRINDL as detailed in published protocols [8,9,10,11]. De-identified clinical metadata were provided by the Organ Procurement Organizations. Lung development and pathology were documented by a pediatric pathologist (G.D.) including assessment of alveolarization by radial alveolar counts (RAC) from the RLL (average 6–7 blocks per case) as described previously [12,13]. Hearts were sliced from apex to base; a mid-ventricular slice was utilized for mitochondrial study.

### 2.5. Isolation of Mitochondria

Mitochondria were isolated from freshly obtained heart tissue from the COXPD8 infant by differential centrifugation at 500 and 10,000× *g* [14]. Briefly, the tissue was washed in ice-cold PBS and then with isolation buffer (225 mM Mannitol, 70 mM Sucrose, 20 mM Tris pH 7.2, 0.5 mM EDTA and 0.5 mM EGTA). The tissue was minced and homogenized with an Elvehjem potter. The final sediment was resuspended in a small volume of EDTA- and EGTA-free isolation buffer. Left ventricular mitochondria were also isolated from frozen tissue from all heart samples and stored frozen at −80 °C, using the same protocol except that mitochondria were sedimented at 12,000× *g*.

### 2.6. Oxygen Consumption

Mitochondrial oxygen consumption was performed with a Clark-type oxygraph (Hansatech, King’s Lynn, UK, PPI Systems), as described [14]. Respiration was induced by the addition of 3 mM malate/5 mM glutamate followed by the addition of 1 mM ADP and followed by 10 mM succinate.

### 2.7. Electrophoresis and Immunoblotting

Native and denaturing polyacrylamide gel electrophoresis (PAGE) was performed, as published [15,16]. Per lane 20 µg of protein was used. For native PAGE, samples were solubilized with digitonin (5 µg/µg protein) on ice for 20 min. Gels were either used for in-gel assays (IGA) [16] or proteins were transferred onto nitrocellulose membranes using a Trans-Blot Turbo Transfer System (Biorad) [17]. Membranes were then blocked with Biorad Blocking reagent and incubated with primary antibodies (Table 2) dissolved in 3% bovine serum albumin in Tris-buffered saline followed by fluorescent tag labeled secondary antibodies (Starbright 520 and Starbright 700 from Biorad; dilution 1:10,000). Signals were detected using a ChemiDoc station from Biorad. Gels and immunoblot images were processed using Image Lab (version 6.1, Research Resource Identifier SCR_014210 Biorad, Hercules CA) and Adobe Photoshop (version 26.8.1, San Jose CA and Illustrator (version 29.7.1, San Jose CA). Densitometry was determined using Fiji/Image J.

### 2.8. Enzyme Assays

The enzymatic activity of the ETC complexes was assessed, as described [18,19]. Cx I activity was measured as NADH-ubiquinone dehydrogenase and by its ability to transfer electrons to Cx III (NADH-cytochrome c oxidoreductase). Where necessary, the assay was repeated with 2 mg/mL rotenone to assess Cx I specific, rotenone-sensitive activity. Cx II was measured by its ability to oxidize DCPIP and by its ability to transfer electrons to Cx III (succinate-cytochrome c oxidoreductase). Cx IV activity was assessed by the oxidation of reduced cytochrome c (ab140219, Abcam). Cx V was measured by its reverse function to hydrolyze ATP in the absence or presence of oligomycin. Citrate synthase was measured by the generation of citrate from oxaloacetate and acetyl-CoA in a phosphate-buffered saline containing 0.01% Triton X-100. Lactate dehydrogenase (LDH) was measured by its ability to reduce pyruvate in the presence of NADH.

## 3. Results

### 3.1. Sample Processing

Following recovery, the organs from the COXPD8 patient were placed in cold histidine-tryptophan-ketoglutarate transplantation buffer, shipped on wet ice and received in the LungMAP laboratory approximately 34 h after death with recorded 3 h of warm ischemic time and 31 h of cold ischemic time. The comparison case organs were recovered similarly (Table 1). The whole heart weight was 73.4 g (Table 3, >95th percentile) [20]. The histologic appearance of the lung was immature for age and diminished RAC supported pulmonary hypoplasia (Table 3 [12]).

**Figure 1:**
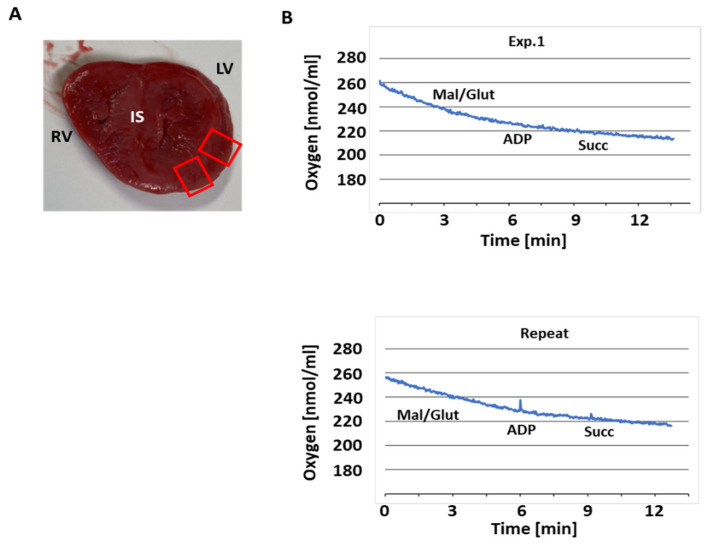
Mitochondrial respiration in COXPD8 mitochondria. (**A**) Photo of a section of heart tissue of the patient with COXPD8. Red boxed areas indicate LV wall used to isolate mitochondria. RV and LV: right and left ventricle, both measured approximately 1 cm thick; IS interventricular septum, measured approximately 1.5 cm thick. (**B**) Original oxygraphy recordings show that isolated LV mitochondria did not respond to substrates (ADP: adenosine diphosphate; Mal/glut: malate/glutamate; Succ: succinate).

A transverse section of the heart (Figure 1A) was cut into pieces of approximately 0.2–0.3 g. Mitochondria were immediately isolated from one piece of the left ventricle to assess mitochondrial respiration and other functional parameters of the ETC. The remaining tissue was quickly frozen at −80 °C. To confirm the results of the initial experiments, a second piece of heart tissue was thawed after 2 weeks and processed together with previously frozen tissue specimens from the 5 comparison patients.

### 3.2. Respiratory Activity

Freshly isolated heart mitochondria (≈115 µg protein) were resuspended in respiration buffer and stimulated with malate/ glutamate (basal respiration, V0), and followed by the addition of ADP (maximal respiration, Vmax, Figure 1B). No oxidative activity was observed. To exclude the possibility of an inactive Cx I [21,22], 10 mM succinate was added to stimulate Cx II mediated respiration. However, while oxygen in the measuring vessel decreased in two trials, no response to the substrates (malate/glutamate and succinate) or ADP was detectable. This indicated severely compromised mitochondria, as we have previously measured respiratory activity in similar samples [23].

### 3.3. ETC Complex and Supercomplex Assembly

**Figure 2:**
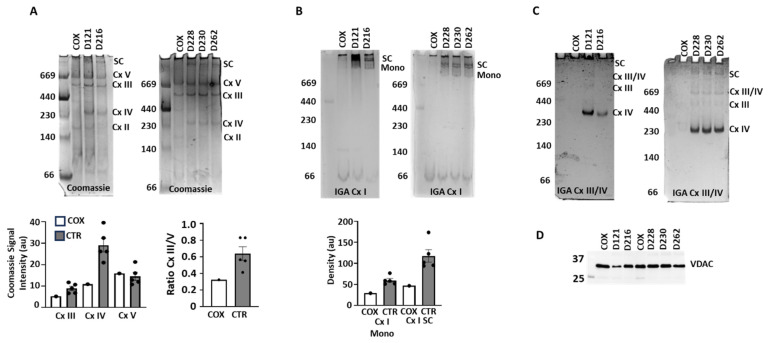
Clear native electrophoresis indicates the absence of Cx I and IV in the COXPD8 patient. (**A**) Coomassie stain of the COXPD8 and 5 unrelated comparison heart tissues and densitometric analysis in the bottom panel. (**B**) In-gel assays (IGA) for Cx I shows severely decreased NADH oxidase activity in the COXPD8 patient. Bottom panel: Densitometric analysis of the monomer (Mono) of Cx I and supercomplexes containing Cx I. (**C**) IGAs for detection of Cx III and Cx IV activity. (**D**) An aliquot of each sample used for native electrophoresis was analyzed by SDS electrophoresis and labeled for VDAC as housekeeping protein. 20 µg protein was loaded in each lane. COX: COXPD8 patient, CTR: control patients, Mono: monomer, SC supercomplexes.

To investigate why the mitochondria of this patient showed no respiratory activity, we examined the protein complexes of the ETC by clear native electrophoresis (Figure 2). Coomassie staining (Figure 2A) showed lower or absent levels of Cx III and Cx IV and supercomplexes (SC), but no change in Cx V in the COXPD8 patient relative to comparison samples (Figure 2A, bottom panels). The ratio of Cx III/V indicates less Cx III in the COXPD8 patient. The staining of Cx II and the monomer of Cx I were too faint for analysis in all samples investigated.

In-gel assays (IGA) to visualize the activities of Cx I, III and IV confirmed the absence or severe reduction in enzymatically active supercomplexes in COXPD8 mitochondria compared to controls (SC in Figure 2B,C). Monomeric Cx I was very low in the COXPD8 heart but also in all control hearts (Figure 2B). Instead, most of Cx I activity was present in supercomplexes in control hearts (Figure 2B) but was absent in COXPD8 mitochondria. Similarly, Cx III and Cx IV activity, either as active monomers or in supercomplexes, was absent in COXPD8 mitochondria, but not in control hearts except in patient D216. In this patient the IGA activity of Cx IV appeared to be lower and the molecular weight of the active Cx IV was slightly lower compared to other control hearts (Figure 2C). Protein loading was similar for each lane (Figure 2D), suggesting that the absence of Cx I, III, and IV in the COXPD8 heart and the lower Cx I IV activity in patient D216 was not due to loading irregularities.

**Figure 3:**
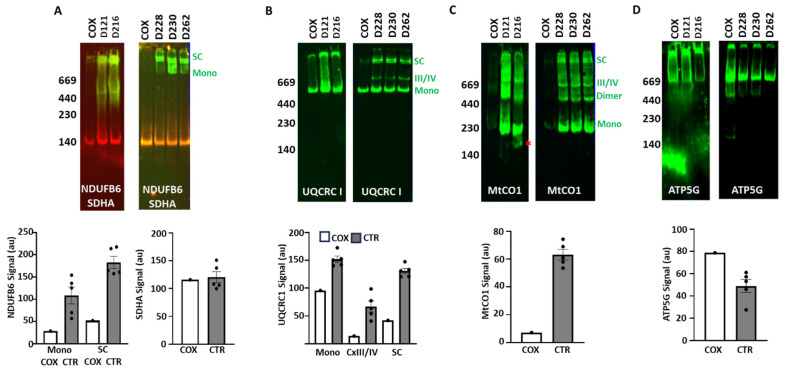
Immunoblotting after clear native electrophoresis confirms the absence of Cx I and Cx IV. (**A**) Detection and analysis of Cx I subunit NDUFB6 expression (in green) confirms absence of monomeric Cx I and almost no supercomplexes in the COXPD8 patient, while the detection of Cx II subunit SDHA (in red) was similar in all samples. (**B**) The monomer of Cx III was detected in the COXPD8 patient, but supercomplexes were not when labeled for UQCRC1). (**C**) Faint detection of Cx IV subunit MtCO1 in monomers and supercomplexes in COXPD8 patient compared to controls. An additional signal for MtCO1 at ≈200 kD in patient D216 is indicated by *. (**D**) Detection of the Cx V subunit ATP5G was not affected by COXPD8. 20 µg protein was loaded in each lane of these 4–10% native gels. VDAC detection for loading control is presented in Figure 2D. COX: COXPD8 patient, CTR: control patients, mono: monomer, SC: supercomplexes.

We then validated the IGA results by immunoblotting to detect functionally active and inactive ETC complexes and supercomplexes. Consistent with the IGA, the Cx I subunit NDUFB6 was present in COXPD8 mitochondria, but at much lower levels in supercomplexes and barely detectable as a monomer (Figure 3A). Cx II subunit SDHA was detected in all samples and showed no differences in the COXPD8 patient (Figure 3A). The Cx III subunit UQCRC1 was detected as a monomer in all samples (Figure 3B) but was slightly lower in the COXPD8 patient. However, supercomplexes, containing Cx III together with Cx I and/or Cx IV were low or absent in the COXPD8 patient (Figure 3B,C). Consistent with this result, the subunit MtCO1 of Cx IV was barely detected in monomers and supercomplexes in COXPD8 mitochondria (Figure 3C). In addition, this antibody shows a different pattern of bands in control patient D216, where the monomer and dimer of Cx IV had a slightly lower molecular weight, and an additional smaller band of about 200 kD was also observed (* in Figure 3C). Detection of ATP5G, a protein of the membrane-embedded portion of Cx V, appeared to be slightly elevated in the Cx V monomer in the COXPD8 patient (Figure 3D).

### 3.4. Protein Expression

**Figure 4:**
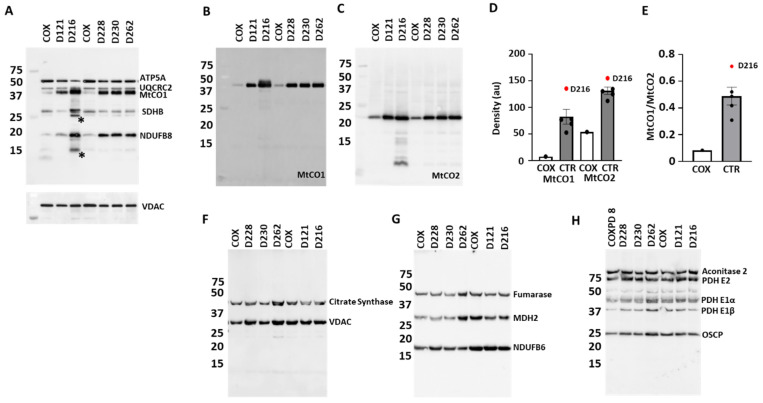
SDS electrophoresis of mitochondria from COXPD8 and control hearts. (**A**): Immunoblotting shows the expression of a subunit of every ETC complex relative to VDAC expression. (**B**): Expression of the Cx IV subunits MtCO1 and MtCO2 in COXPD8 (COX) and control (CTR) patients. (**C**–**E**): Expression of Cx IV subunits MtCO1 and MtCO2 was lowest in COXPD8 patient and highest in patient D216 (●). (**F**–**H**): Expression of various TCA cycle enzymes was not affected in the COXPD8 patient. 5 µg protein were separated per lane on a 15% SDS gel. COX: COXPD8 patient, CTR: control patients, MDH2: malate dehydrogenase 2; PDH: pyruvate dehydrogenase; OSCP: oligomycin sensitivity conferring protein.

Immunoblotting after denaturing/SDS electrophoresis showed lower expression of the Cx I subunit NDUFB8 (Figure 4A), while the labeling for NDUFB6 remained unchanged (Figure 4G) in the COXPD8 patient. Labeling for Cx II (SDHB) and Cx V (ATP5A) was slightly higher and Cx III (UQCRC2) was unchanged in the COXPD8 patient (Figure 4A). Labeling for Cx IV subunits MtCO1 and MtCO2 was lower in the COXPD8 patient (Figure 4A–D). The ratio of the signal density of MtCO1 to MtCO2, both subunits of the catalytic core of Cx IV, indicates a more severe impact on the expression of MtCO1 due to the AARS2 mutation (Figure 4E).

The D216 heart showed stronger signals for NDUFB8, SDHB, MTCO1 and MtCO2 but no change in ATP5A and UQCRC2 labeling compared to other control/comparison patients (Figure 4A–E). The OXPHOS antibody cocktail labeled 2 proteins (*) in this patient that were not seen in the other controls (Figure 4A). These proteins could be truncated versions or degradation products of any of the proteins the antibody cocktail was able to recognize. In addition, the MtCO2 antibody detected several MtCO2 fragments in patent D216. Detection of the house keeping protein VDAC did not show any differences (Figure 4F), indicating that differences in the expression of the ETC proteins in D216 were not due to unequal protein loading or mitochondrial mass.

The expression of the several enzymes of the tricarboxylic cycle (aconitase, citrate synthase, fumarase, malate dehydrogenase and pyruvate dehydrogenase) were unchanged in the COXPD8 patient compared to the control patients (Figure 4F–H), supporting reports that AARS2 mutations lead to highly specific phenotypes [24].

### 3.5. Enzymatic Activity

**Figure 5:**
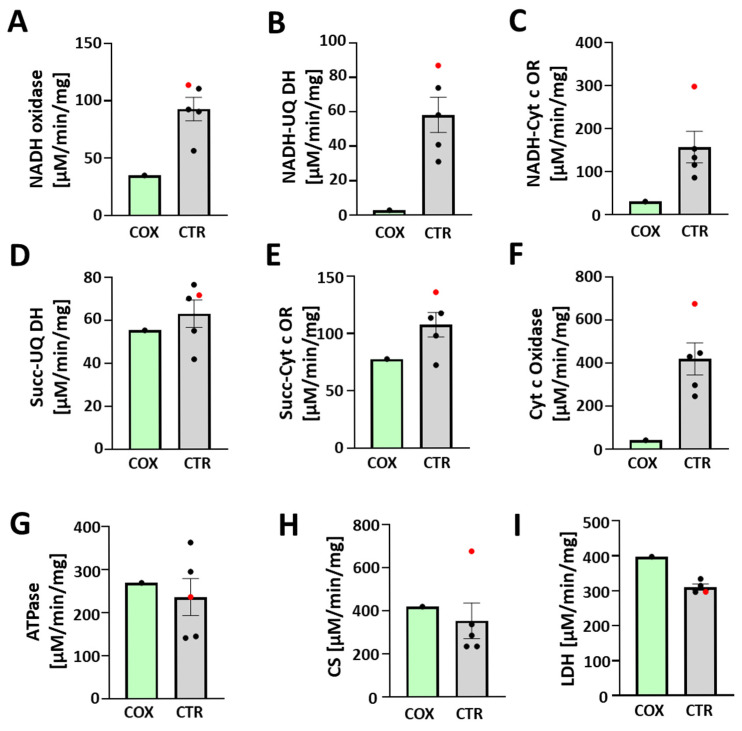
Enzymatic activity of ETC complexes, citrate synthase, and lactate dehydrogenase. Enzymatic activity of ETC Cx I-V (**A**–**G**), citrate synthase (**H**), and lactate dehydrogenase (**I**). Enzymatic activities of patient D216 are highlighted in red (●). All activities are presented in µM/min/mg. NADH-UQ DH: NADH-ubiquinone dehydrogenase; NADH-Cyt c OR: NADH-cytochrome c oxidoreductase; Succ-UQ DH: succinate-ubiquinone dehydrogenase; Succ-Cyt c OR: succinate-cytochrome c oxidoreductase; Cyt c Oxidase: cytochrome c oxidase; ATPase: oligomycin-sensitive ATPase activity of Cx V; CS citrate synthase; LDH lactate dehydrogenase.

The absence of functional ETC complexes I and IV in the COXPD8 patient was also apparent when we measured enzymatic activities (Figure 5). Activities related to Cx I (NADH oxidase, NADH-ubiquinone dehydrogenase, NADH-cytochrome oxidoreductase) and Cx IV (cytochrome c oxidase) were decreased in the COXPD8 patient compared to controls (Figure 5A–C,F). The activities of Cx II (succinate-ubiquinone dehydrogenase, succinate-cytochrome c oxidoreductase) and Cx V (oligomycin-sensitive ATPase activity) were not affected compared to control patients (Figure 5D,E,G). Citrate synthase activity, a commonly used marker for mitochondrial function and biogenesis (Figure 5I); [25,26], was also not different in heart mitochondria of the COXPD8 patient, confirming a highly specific impact on Cx I and IV. Lactate dehydrogenase (LDH, Figure 5I) activity was increased in the COXPD8 patient, likely indicating more reliance on anaerobic glycolysis for ATP production.

In addition, among the control patients, the activity of Cx IV for patient D216 was the highest (Figure 5F, red), consistent with the highest protein expression for MtCO1 and MtCO2 by immunoblotting. Cx I activity was also highest in this patient (Figure 5A–C, red), particularly if measured as NADH-cytochrome c oxidoreductase activity. The activity of the citrate synthase was also very high in this patient and considered an outlier by Dixon’s Q test among the control patients (Figure 5H), further supporting the possibility of an undiagnosed underlying mitochondrial condition in patient D216.

## 4. Discussion

Mutations that impact the expression of mitochondrial proteins encoded on the mitochondrial or nuclear DNA are often complex [27] and currently no cure is available. The prevalence of clinically affected adults with mitochondrial diseases caused by mitochondrial and nuclear DNA mutations is about 12.5 in 100,000 [27,28], but with advancing technology, it has become apparent that the majority of cases of mitochondrial disorders in children result from nuclear DNA mutations. In 1998, Lamont et al. demonstrated that mitochondrial DNA mutations account for <10% of all mitochondrial disorders in children [29,30].

The COXPD8 patient presented in this report had one likely pathogenic mutation and one variant of unknown significance of the nuclear encoded AARS2 gene, and his phenotype was consistent with this syndrome. Despite the standard non-specific, “mitochondrial” therapy, the patient passed away in the neonatal period with primarily cardiac and respiratory insufficiency. Evaluation of the heart post-mortem confirmed marked hypertrophy with increased weight and concentric ventricular wall and septal thickness. The lung pathology was consistent with pulmonary hypoplasia with reduced radial alveolar count (Table 3) and immature lung architecture (double-capillary loop) for age. AARS2 mutations affect a small number of patients, but they are associated with intriguing tissue- and cell-specific phenotypes that typically involve the central nervous system [4]. A possible explanation of AARS2-specific disease phenotypes may be attributable to the location of the pathogenic variants in the protein and possible posttranslational modifications [4].

From a molecular standpoint, we found that the mutations of the AARS2 gene(s) prevented the assembly of functionally active Cx I and Cx IV of the ETC; monomers of these complexes were almost absent and consequently, and there was a severe decrease in respirasome supercomplexes containing Cx I, III, and IV. The presence of a small amount of Cx I/III/IV supercomplexes but almost no Cx I monomers may be due to protection of these monomers by their immediate incorporation into supercomplexes as has been observed with NDUFS4 deleted in the mouse heart [31]. ETC supercomplexes remodel the inner membrane cristae and are thought by some to promote OXPHOS efficiency and prevent ROS production, and their loss in the index patient may have played a role in bioenergetic dysfunction and caused continuous damage to this patient’s mitochondria.

Our data confirm published data on the specificity of the c.2872 C>T mutations towards a dysfunction of Cx I and IV [6]. The c.1738 C>G mutation has not been described before, and our data indicate that, with respect to assembly and function of Cx I and Cx IV, it may have worsened the effect of the c.2872 C>T mutation. These results also explain the complete absence of a respiratory response through either Cx I or Cx II of the ETC, even though we have shown previously that respiratory activity can be measured in specimens obtained in this manner [23]. Furthermore, there were no apparent differences in the expression or activity of tested TCA cycle enzymes, which are encoded in the nucleus, but there was an increase in LDH activity. Overall, this suggests that the absence of an intact ETC, despite the presence of an intact TCA cycle led to reliance on anaerobic glycolysis in this patient. It is also possible that the intact TCA cycle allowed for anabolic production of metabolites to permit myocyte hyperplasia and/or hypertrophy.

The control/comparison hearts used in these assays were a random sampling of a limited number of hearts received by the LungMAP program. While comparing results of this COXPD8 patient to age-matched patients, we found that patient D216 was an outlier who might also have had a mitochondrial defect. We found a decreased enzymatic activity of Cx IV in in-gel assays, but a higher activity of Cx IV in a spectrophotometric assay, an increased expression of the Cx IV subunits MtCO1 and MtCO2, a lower molecular weight of the Cx IV monomer in native gels, and additional MTCO2 proteins in denaturing gels. Together this suggests an underlying defect in the assembly of Cx IV that is not seen in the other ETC complexes. Of note, the D216 patient presented with a 1-month history of wet cough and acute, profound and persistent metabolic acidosis (base deficit −29 to −12 over the 36 h hospitalization, lactate not available) in association with viral respiratory tract infection. Postmortem heart weight was approximately 95% for age but body weight was also in the 90th percentile. Although it is possible that this patient’s mitochondrial dysfunction was due to the viral infection, we think it likely that there was an underlying mitochondrial disorder.

These results highlight the clinical severity of COXPD8 syndrome and other mitochondrial disorders. Although COXPD8 syndrome is not associated with congenital heart defects, our patient and others reported in the literature have infantile hypertrophic cardiomyopathy, so this disease should be considered in newborns with cardiac hypertrophy not explained by other mechanisms such as maternal diabetes. Severe pulmonary hypoplasia also supports a mitochondrial role in normal lung development and growth. Furthermore, our control patient D216 had an undiagnosed and unsuspected mitochondrial disorder that does not appear associated with cardiac hypertrophy nor pulmonary hypoplasia, and it remains unclear if the month-long wet cough and prolonged metabolic acidosis seen in this patient was due to this mitochondrial defect. Although we do not know full clinical details of this patient, these finding highlights that the frequency of mitochondrial disorders is probably higher than currently recognized in neonates. Together, these data document the clinical events occurring in a case of nuclear mitochondrial gene mutations. It is not uncommon for mitochondrial disorders in neonates to appear as suspected cases of hypoxic–ischemic encephalopathy due to the hypotonia and cardiorespiratory failure at birth causing low APGAR scores and metabolic acidosis, often with pulmonary hypertension. This case reminds clinicians to have a higher index of suspicion for mitochondrial disease in neonates with unexplained cardiomyopathy especially when associated with hypotonia, respiratory failure and seizures. Finally, understanding the etiology of mitochondrial disorders will allow targeted therapy, and new modalities for such are under current investigation.

## 5. Conclusions

In conclusion, our analysis demonstrates that the COXPD8 syndrome in this patient is associated with a failed assembly of Cx I and IV of the ETC in the presence of two mutations of the AARS2 gene. Our data are consistent with previous human subjects with this disease [1,2], but it is rare to be able to test these mechanisms in freshly isolated mitochondria for human cardiac tissue. In addition, when we evaluated mitochondrial activity in several available samples as controls, we found that one other sample (D216) had a defect in Cx IV function and protein expression that was different from our proband. The genetic etiology for the second case is unknown.

## Figures and Tables

**Table 1:** Summary of the patients used in this study. Tissues were recovered between 2017 and 2023.

ID	Gestational Age (Weeks)	Sex	Age at Death (Days)	Weight (Percentile)	Cause of Death	WIT/CIT * (Hours)
D228	37	M	1	27	Bladder outlet obstruction, pulmonary hypoplasia, hypertensive changes in pulmonary arteries	2/21
D262	33	M	1	13	Anencephaly, normal lung	3/19
D230	37	M	10	<3	Anencephaly, normal lung	4/27
D121	40	F	35	<3	Hydranencephaly due to in utero vascular accident, normal lung	6/20
D455	38	M	51	5	COXPD8, pulmonary hypoplasia, hypertensive changes in pulmonary arteries	3/31
D216	36	M	157	90	Neonatal abstinence, NICU × 11 days; + respiratory virus, severe and persistent metabolic acidosis, normal lung with patchy lymphocytic inflammation	0/30

* WIT: warm ischemic time; CIT: cold ischemic time.

**Table 2:** Summary of antibodies used.

Antibody	Dilution	Source	Catalogue Number #	Comments
Aconitase 2	1:1000	Thermo Fisher Scientific (Waltham, MA, USA)	PA5-29960	
ATP5G	1:2000	Abcam (Hong Kong, China)	Ab180149	
Citrate Synthase	1:1000	Cell Signaling Technology (Danvers, MA, USA)	14309	
Fumarase	1:500	Thermo Fisher Scientific	PA5-82899	
MtCO1	1:2000	Thermo Fisher Scientific	459600	
MtCO2	1:2000	Thermo Fisher Scientific	MA5-57085	
NDUFB6	1:1000	Abcam	Ab110244	
OxPhos Cocktail	1:1000	Abcam	Ab110413	Mixture of anti-NDUFB8, SDHB, UQCRC2, MtCO1, and ATP5A
PDH	1:1000	Thermo Fisher Scientific	456799	Mixture of anti-PDH E1β, PDH E1α, PDH E2 and OSCP
SDHA	1:4000	Thermo Fisher Scientific	459200	
UQCRC1	1:1000	Thermo Fisher Scientific	459140	
VDAC	1:2000	Abcam	Ab154856	

**Table 3:** Morphological heart and lung measurements.

ID	Heart Weight Observed (g)	Heart Weight Expected (g) 50%ile (5–95%ile)	RAC	Expected RAC
D228	17.3	20 (13–30)	3.3	4
D262	10.7	20 (13–30)	3.2	3
D230	7.5	22 (15–32)	4.2	4
D121	17.9	26 (19–36)	5.0	5
D455	73.4	28 (20–38)	3.5	5–6
D216	49.4	38 (28–48)	7.9	7–8

## Data Availability

The original contributions presented in this study are included in the article. Further inquiries can be directed to the corresponding author(s).

## Abbreviations

The following abbreviations are used in this manuscript:

AARS2	Mitochondrial alanyl-tRNA synthetase gene
BRINDL	BioRepository for Investigation of Neonatal Diseases of the Lung
COXPD8	Combined oxidative phosphorylation deficiency type 8
Cx	ETC complex
DOL	Day of life
ECMO	Extracorporeal membrane oxygenation
ETC	Electron transport chain
g	Gram
HIE	Hypoxic–ischemic encephalopathy
IGA	In-gel assay
LDH	Lactate dehydrogenase
OXPHOS	Oxidative phosphorylation
PAGE	Polyacrylamide gel electrophoresis
RAC	Radial alveolar counts
SC	Supercomplexes
TCA	Tricarboxylic cycle
V_0_	Basal respiration
V_max_	Maximal respiration
